# Serum-to-urine renalase ratio and renalase fractional excretion in healthy adults and chronic kidney disease patients

**DOI:** 10.1186/s12882-020-01737-5

**Published:** 2020-03-04

**Authors:** Natalia M. Serwin, Magda Wiśniewska, Elżbieta Cecerska-Heryć, Krzysztof Safranow, Edyta Skwirczyńska, Barbara Dołęgowska

**Affiliations:** 1 Department of Laboratory Medicine, Pomeranian Medical University, Szczecin, Poland; 2 Department of Nephrology, Transplantology and Internal Diseases, Pomeranian Medical University, Szczecin, Poland; 3 Department of Biochemistry and Medical Chemistry, Pomeranian Medical University, Szczecin, Poland; 4 Department of History of Medicine and Medical Ethics, Pomeranian Medical University, Szczecin, Poland

**Keywords:** Chronic kidney disease, Glomerular filtration rate, Renalase, Renal markers

## Abstract

**Background:**

Renalase is a flavoprotein that plays a protective role in chronic kidney disease (CKD) and cardiovascular diseases. The secretion and way of action of this protein are still discussed. The aim of our study was to estimate the balance between serum and urine renalase in healthy individuals and CKD patients, using two parameters: fractional excretion (FE) and serum-to-urine renalase ratio (StURR).

**Methods:**

Our study involved 28 healthy volunteers and 62 patients with CKD in stages I to IV. The concentration of renalase in serum and urine was measured using an enzyme-linked immunosorbent assay (ELISA) kit (EIAab, Wuhan, China). We analyzed associations between renalase levels in urine and serum, and other parameters: sex, age, GFR, presence of hypertension, diabetes, and proteinuria, and determined the serum-to-urine renalase ratio and fractional excretion of renalase.

**Results:**

Renalase and serum-to-urine ratio were significantly higher in CKD patients in comparison with the control group. Fractional excretion was lower in CKD patients but this difference did not reach the statistical significance (*p* = 0.092). Multivariate analysis performed in the CKD group showed, that from mentioned parameters, serum renalase was the only significant independent factor strongly positively associated with urinary renalase concentration.

**Conclusions:**

The serum-to-urine ratio is significantly and about 6.5-fold higher in CKD patients, and the fractional excretion of renalase is 3-fold, but not significantly lower in CKD patients. Renalase levels in both serum and urine are not related to the glomerular filtration rate and not associated with blood pressure.

## Background

Renalase is a soluble flavoprotein of the molecular mass of 37.8 KDa, found in blood and urine of humans, rats, and mice [1–4]. Studies on animal models of chronic kidney disease (CKD) show, that renalase prevents or reduces damage and necrosis of kidney tissues, and has hypotensive and cardioprotective properties [5–7]; also in vitro analysis of acute kidney injury (AKI) mechanisms indicates, that this protein diminishes kidney damage [8, 9].

Knowledge about the **concentration** of renalase in blood and urine of healthy subjects and patients with chronic kidney disease, or animal models of CKD, is not extensive and shows some discrepancies. Most of results show, that concentration of renalase in serum of subjects with CKD, hemodialyzed, renal transplant recipients or patients with coronary artery disease (CAD) is higher when compared to healthy subjects [1, 10–12], while one Western blot study performed on human blood plasma showed a decrease in concentration of this protein in end-stage renal disease (ESRD) [1]. Less information is available on urinary excretion of renalase, however, the centile charts for normalized urinary renalase excretion for children and adolescents were recently evaluated and published [13].

The results of studies on renalase potential enzymatic **activity** did not allow to identify the parameters of reaction specific for this protein. Initially, it was suggested that renalase deaminates biogenic amines in a monoamine oxidase-like pathway, and this reaction results in the formation of hydrogen peroxide [1]; what is more, the concentration of H_2_O_2_ is still used by some researchers as an indicator of renalase activity. Because of the emerging problem and non-specific products of such reaction, other research groups assumed that renalase, in presence of superoxide anion, oxidates catecholamines to the corresponding aminochromes, e.g.: adrenaline to adrenochrome, noradrenaline to noradrenochrome, dopamine to dopaminochrome [14]. There are also reports indicating that RNLS can be an anomerase converting α-NAD(P) H to β-NAD(P) H [15]. The latest reports suggest that this protein has a protective effect on the kidney irrespective of its enzymatic activity, but by influencing the MAP kinases (MAPK) pathway and preventing kidney damage during acute kidney injury [8].

Our study aimed to evaluate the balance between circulating blood serum renalase and its urinary excretion to determine the serum-to-urine renalase ratio (StURR) and fractional excretion (FE) of renalase in healthy adults and chronic kidney disease patients. We also evaluated the urinary renalase/creatinine ratio in both groups and analyzed the associations between renalase and some parameters which were previously described as ones that have an impact on its concentration: age, sex, GFR, hypertension, diabetes, and proteinuria in the CKD group.

## Methods

### Subjects

The study involved 90 subjects: 28 healthy people (19 women and 9 men) as the control group, and 62 patients (36 women and 26 men) with diagnosed chronic kidney disease (the CKD group) in stages I to IV, according to Kidney Disease Outcomes Quality Initiative (KDOQI): 17 patients with stage I, 16 with stage II, 20 with stage III and 9 with stage IV. The control group consisted of healthy Pomeranian Medical University workers and students. The participants did not report any health problems and did not take any medications. All participants gave written informed consent. The CKD group consisted of patients of the Department of Nephrology, Transplantology and Internal diseases of Pomeranian Medical University in Szczecin. None of the participants was on renal replacement therapy. 13 of the patients were diagnosed with autosomal dominant polycystic kidney disease (ADPKD), 28 with glomerulonephritis, 4 with kidney stones, 4 with primary hypertension that caused the loss of kidney function, 2 patients with type 1 diabetes mellitus, 1 person with type 2 diabetes mellitus, and ten patients with other kidney diseases: systemic lupus erythematosus, kidney donor, pyelonephritis, after nephrectomy, kidney atrophy, two patients with recurrent urinary tract infection and three patients with chronic kidney disease of unknown etiology.

### Material

Blood and urine were collected in the morning on empty stomach, in the shortest possible time interval to give the most accurate information about momentary serum-to-urine renalase balance. Whole blood was drawn into S-Monovette tube (Sarstedt, Numbrecht, Germany) with a clotting activator and left for 30 min at room temperature. Serum was obtained by centrifugation of the clotted blood (10 min, 1000 x g, room temperature). Urine was collected into dedicated sterile urine containers and centrifuged for 20 min (600 x g, room temperature). Both materials were then transferred to smaller tubes and kept in the refrigerator at − 80 °C until use. Since this study did not involve 24-h urine collection, the concentration of urinary renalase was also normalized by comparison to urinary creatinine.

### Assays and calculations

The concentration of creatinine in blood and diluted urine was measured using a ready reagent kit (BioMaxima, Lublin, Poland), basing on Jaffe’s kinetic method, and calculated according to the manufacturer’s instructions. The concentration of renalase both in serum and urine was measured using a commercially available immunochemistry test (ELISA, goat polyclonal; EIAab, Wuhan, China).

The eGFR was calculated using the Modification of Diet in Renal Disease (MDRD) equation: 175 × (SCr)–1.154 × (age)–0.203 × 0.742 [if female] × 1.210 [if black], where SCr (standardized serum creatinine) = mg/dl, age = years. The fractional excretion of renalase (FE_Rnls_) was calculated using the following equation: FE_Rnls_ = (urinary renalase x serum creatinine) / (serum renalase x urinary creatinine) × 100. Serum-to-urine renalase ratio (StURR) was calculated using the following formula: StURR = serum renalase (ng/ml) / renalase in urine (ng/ml). The renalase/creatinine (renalase/Cr) ratio was calculated basing on their concentration in 1 ml of urine (ng of renalase/mg of creatinine).

### Statistical analysis

Statistical analysis was performed using STATISTICA 12.0 PL software. The distribution of variables was determined using the Shapiro-Wilk W test. Since all quantitative data have distribution significantly different from a normal distribution (*p* < 0.05), they are shown as median (upper quartile – lower quartile) and compared using the U-Mann Whitney test when comparing two groups or Kruskal-Wallis ANOVA for more than 2 groups. Correlations were measured using the non-parametric Spearman rank correlation test. For qualitative data, Fisher exact test was used. Multivariate analysis was performed using a general linear model (GLM) with concentrations of renalase and its fractional excretion transformed logarithmically to normalize their distributions.

## Results

Patients characteristics and results of biochemical and ELISA measurements are shown in Table 1. Among 62 CKD patients, 52 had associated hypertension, 19 had proteinuria (< 5 g/24 h urine collection), 1 had nephrotic syndrome (proteinuria > 5 g/24 h urine collection), and 10 had diabetes. 6 of patients had diagnosed cardiovascular diseases or dysfunctions: peripheral atherosclerosis, hypertrophic cardiomyopathy, heart failure, state after myocardial infarction and 2 cases of coronary artery disease (CAD).

**Table 1:** Basic biochemical and immunochemical analysis and differences between control group and chronic kidney disease patients. Quantitative data are shown as median (lower quartile - upper quartile)

	Control	CKD patients	***p*** value
***N*(males/females)**	28 (9/19)	62 (27/35)	0.36
**Age (years)**	48 (27–53.5)	52 (41–61)	0.040
**Systolic blood pressure**	139.5 (127–160,75)	135 (120–140)	0.099
**Diastolic blood pressure**	91.5 (82.25–98.75)	80 (75–85)	< 0.001
**Heart rate**	76 (64.75–85)	75 (70–80)	0.71
**Serum creatinine (mg/dl)**	0.78 (0.69–0.95)	1.44 (1.13–1.92)	< 0.001
**Urine creatinine (mg/dl)**	113.35 (92.23–161.54)	74.87 (50.21–107.94)	< 0.001
**Proteinuria (g/24 h)**	–	1.5 (0.72–2)	–
**eGFR (ml/min/1.73m**^**2**^**)**	91.5 (79.3–99)	61 (40–91)	< 0.001
**Serum renalase**	11.1 (2.5–26.5)	36.1 (18.3–109.1)	< 0.001
**Urine renalase**	76.4 (26–114)	25.1 (13.8–104.4)	0.095
**Urinary renalase/Cr ratio (ng/mg)**	53.7 (22.7–96.5)	44 (17.7–172)	0.99
**Serum-to-urine renalase ratio**	0.177 (0,023 – 0,94)	1.146 (0.63–1.75)	< 0.001
**Fractional excretion of renalase (%)**	4.68 (0.65–21.22)	1.60 (0.78–3.25)	0.092

In the control group, the diastolic blood pressure (DBP) was significantly higher than in the CKD group. There was no significant difference in systolic blood pressure (SBP). Since elevated arterial pressure was not a criterium for excluding patients from the study, and at the same time appeared to be scientifically significant in terms of renalase concentration, all results were also subjected to statistical analysis. As with patients with CKD, no relationship between renalase concentration and diastolic or systolic pressure was observed. Serum renalase did not correlate with any other parameter. The same observation was made for creatinine. Concerning the urinary renalase/Cr ratio, it correlated moderately negatively with the heart rate (Rs = − 0.48, *p* = 0.010). Heart rate correlated also negatively with urinary renalase (Rs = − 0.40, *p* = 0.035), and positively with urinary creatinine (Rs = 0.47, *p* = 0.011). There was no correlation between FE and any other parameters (SBP, DBP, HR).

The concentration of renalase in the serum of CKD patients was much higher in comparison to material from healthy individuals. An inverse tendency was observed in urinary renalase values, where excretion of this protein in the CKD group was moderately, but not significantly, lower than in the control group. Urinary renalase/creatinine ratio (ng/mg) did not differ between groups. The FE_Rnls_ values were higher in healthy adults than in CKD patients, however, this difference did not reach the statistical significance (*p* = 0.09).

There were no significant associations (*p* > 0.05, Mann-Whitney test) between any of renalase parameters and qualitative parameters of CKD group (sex, presence of hypertension, cardiovascular disease, diabetes, and proteinuria). Only borderline significant associations of the presence of proteinuria with renalase FE (positive, median 0.030 vs 0.013, *p* = 0.077) and StURR (negative, median 0.76 vs 1.27, *p* = 0.051) were observed. A comparison of renalase levels between patients in different stages of CKD also shown no significant differences (*p* = 0.90, Kruskal-Wallis ANOVA). Median (lower quartile – upper quartile) values of renalase for each stage were: I: 34.73 (20.71–129.7), II: 41 (18.11–104), III: 32.29 (14.15–106.35), IV: 37,67 (26.6–85.77).

**Fig. 1:**
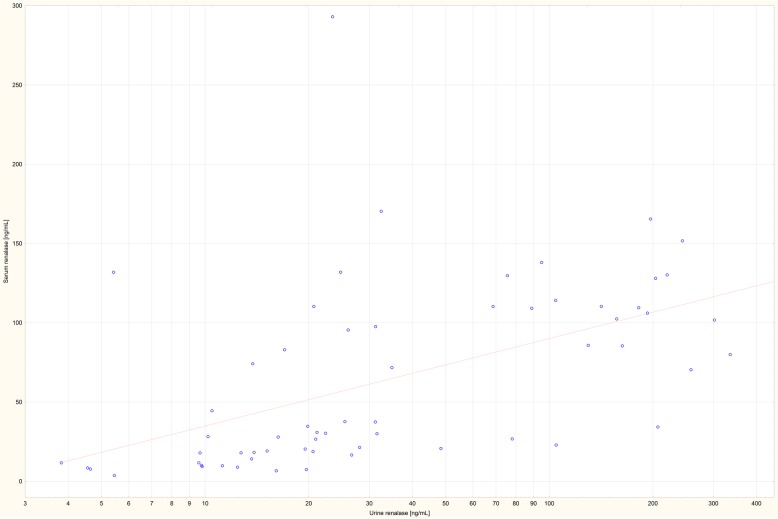
Correlation between serum and urine renalase in chronic kidney disease patients

Spearman rank correlation coefficients are shown in Table 2. This analysis showed no significant correlation between eGFR and StURR among individuals in both analyzed groups. A strong correlation between serum renalase and urine renalase (Rs = 0.66; *p* < 0.001) was observed (Fig. 1).

**Table 2:** The most important correlations coefficients evaluated using Spearman rank correlation test

		Rs	***p***
StURR vs. eGFR	Control	−0.19	0.32
	CKD	−0.01	0.97
StURRn vs. eGFR	Control	−0.20	0.30
	CKD	0.05	0.68
Serum renalase vs. eGFR	Control	−0.18	0.37
	CKD	0.05	0.71
Urine renalase vs. eGFR	Control	−0.10	0.62
	CKD	0.04	0.75
Urine renalase/Cr vs. eGFR	Control	−0.11	0.60
	CKD	−0.03	0.82
Age vs. eGFR	Control	−0.04	0.86
	CKD	−0.31	0.015
Serum renalase vs. urine renalase	Control	−0.07	0.73
	CKD	0.66	< 0.001
Serum renalase vs. proteinuria g/24 h	CKD	−0.22	0.37
Urine renalase vs. proteinuria g/24 h	CKD	−0.23	0.35

Multivariate analysis performed in CKD group with logarithm of urinary renalase concentration as dependent variable and logarithm of serum renalase concentration and six covariates (sex, age, GFR, presence of hypertension, diabetes, and proteinuria) as independent variables, showed that serum renalase was the only significant independent factor strongly positively associated with urinary renalase concentration (standardized β = + 0.67, *p* < 0.001; R^2^ = 0.47, *p* < 0.001 for the whole model) (Table 3).

**Table 3:** General linear model with logarithm of urinary renalase concentration as dependent variable and logarithm of serum renalase concentration and six covariates as independent variables in CKD patients

Independent variables	Standardized β coefficient (95% confidence interval)	p value
Male sex	+ 0.01 (− 0.22 − + 0.23)	0.96
Age	− 0.10 (− 0.32 − + 0.12)	0.35
GFR	+ 0.05 (− 0.18 − + 0.29)	0.65
Hypertension	+ 0.02 (− 0.23 − + 0.26)	0.90
Diabetes	−0.01 (− 0.22 − + 0.20)	0.92
Proteinuria	+ 0.15 (− 0.06 − + 0.37)	0.16
Logarithm of serum renalase concentration	+ 0.67 (+ 0.46 − + 0.87)	< 0.001

R^2^ = 0.47, *p* < 0.001 for the whole model

Similar multivariate analysis in CKD group with logarithm of renalase fractional excretion as dependent variable and six covariates listed above as independent variables did not show any significant association (R^2^ = 0.06, *p* = 0.72 for the whole model), even for proteinuria (β = + 0.20, *p* = 0.16), which showed a borderline positive association with renalase FE in univariate analysis.

## Discussion

Renalase is a small molecule that should be easily filtered by the kidney, however, there is still no consensus regarding the mechanism on which renalase regulation and action is based. As with many other factors, its accumulation may be caused by abnormal production and secretion of this protein, together with limited filtration capacity or impaired filtration due to structural or functional abnormalities of the kidney. Due to the potentially protective effect of renalase, this filtration may be also limited physiologically, or renalase could be reabsorbed under conditions of increased demand for this protein. As already shown in many studies, the elevation of renalase is observed e.g. after acute exercise, when its expression in skeletal muscles increases, and decreases in other tissues indicating that it is a reaction to oxidative stress [16]. A similar observation was made in another study in which renalase blood concentration increased by 3-fold 30 min after epinephrine infusion with a suggestion, that this is due to potential renalase enzymatic properties [17]. Results of some of the scientific research also point out that renalase might be a cytokine related to kidney dysfunction and inflammatory processes affecting CKD patients. As was further shown, an elevation in catecholamines concentration, especially epinephrine, accompanying kidney diseases due to nervous system hyperactivity, stimulates expression of renalase [18]. Many known cytokines are rather poorly excreted in conditions of their much higher production, which is one of the mechanisms involved in higher mortality among patients with acute kidney injury. Elevation of serum renalase may be a defense mechanism against further injury of the kidney. It has been proven that blood renalase might activate Akt kinase, and therefore MAPK kinases pathway, preventing or reducing kidney damage, what was observed in vitro on tissue model of acute kidney injury [8]. What is more, renalase-dependent MAPK signaling and cytoprotection was described as mediated by PMCA4b receptor [19]. *PMCA4b* gene is expressed in many tissues, mainly in endometrium, fat, and skin, but also in heart, urinary bladder, and kidney tissues [20]. This receptor is a Ca2 + −ATPase, responsible for maintaining the electrolyte concentration, but its role in Ca2+ handling in the kidney was not discernible, and its function in renal tissues remains an open question [21]. As our results indicate, serum renalase differs significantly between healthy adults and CKD patients and is much higher in the latter group, and that there is a significant difference in the StUR ratio. At the same time, there is no correlation between serum and urine renalase in healthy individuals, and the multivariate analysis in CKD group shows, that serum renalase is the only significant independent factor strongly positively associated with urinary renalase concentration. Therefore, in physiological conditions, this molecule, like creatinine, is continuously produced and filtered. This finding is supported by fact, that the urinary renalase/Cr ratio is very similar between control and CKD group (medians 53.7 and 44, respectively; *p* = 0.99) indicating, that they are both removed in a balanced proportion. In renal patients, RNLS probably undergoes a “recycling” mechanism in the kidney or there is a barrier preventing from excess loss of this particular protein, even in patients with proteinuria, as it does not influence urinary renalase concentration.

The elevation in serum renalase has been also repeatedly associated with heart and circulatory dysfunctions, very common in the CKD, since heart seems to be another very important source of RNLS. Low concentration of renalase is considered as one of the predictive factors of coronary artery disease (CAD) [8, 12, 22], and an increase in serum renalase is associated with significantly greater hazards of all-cause mortality and adverse renal outcomes [23]. In our study, the presence of hypertension or any of cardiovascular disease had no impact on renalase concentration. What is more, none of the renalase parameters (its concentration in serum and urine, FE, StURR, urinary renalase/Cr) were related to systolic or diastolic blood pressure, nor to heart rate. What is interesting, blood pressure in the control group was higher than in the CKD group, and this association reached the statistical significance in the case of diastolic pressure. Despite this, renalase concentration in the serum of healthy adults was significantly lower, which strongly suggests and indicates that renalase does not have a general antihypertensive effect, described in many scientific reports. It does not mean, that RNLS does not have such properties at all, but probably possess them only under certain conditions.

The urinary renalase/Cr (ng/mg) ratio was firstly described in 2014 and only for healthy children and adolescents [13]. The authors established the reference values and percentiles for urinary renalase excretion showing, that in the youngest children (< 3y.o.) concentration of renalase is significantly higher than in other age groups (3–5.9, 6–8.9, 9–11.9, 12–14.9 and 15–17.9). Urinary renalase/Cr median for the < 3 y.o. children was 245.46 ng/mg, while in the adolescents – 99.51 ng/mg. In our study, this ratio was 53.7 ng/mg in control (age median 48) and 44 ng/mg in the CKD group (age median 52), what is consistent with the assumption that this coefficient decreases with age, although in our research no significant association between age and renalase/Cr was observed.

## Conclusions

We have evaluated the serum-to-urine ratio, which is significantly and about 6.5-fold higher in CKD patients, and the fractional excretion of renalase, which is 3-fold, but not significantly lower in CKD group. Therefore, there must be a mechanism that prevents renalase from escaping through the glomerular filtration barrier in renal patients. Renalase concentration is also unrelated to the glomerular filtration rate and is not associated with blood pressure both in healthy people and CKD patients. Similar research should be performed in cardiac patients without kidney diseases to re-discuss the role of renalase in cardiovascular diseases.

We are aware that our study has some limitations, mainly related to a relatively small group of respondents. Also, the control group consisted of people from the same small environment, and the volunteers from the control group were significantly younger than patients with CKD. At the same time, age does not correlate either with the concentration of renalase or with eGFR, so this fact should not have an important impact on results. The rather limited discussion results from the absence of similar studies and further considerations would be purely speculative.

## Data Availability

The full datasets generated and/or analyzed during the current study are not publicly available due to confidential data, but some of them are available from the corresponding author on reasonable request.

## Abbreviations

CAD: Cardiovascular disease
CKD: Chronic kidney disease
ESRD: End-stage renal disease
FE_Rnls_: Fractional excretion of renalase
RNLS: Renalase
